# Similarity Clustering for Representative Sets of Inorganic Solids for Density Functional Testing

**DOI:** 10.1021/acs.jctc.1c00536

**Published:** 2021-12-17

**Authors:** Péter Kovács, Fabien Tran, Allan Hanbury, Georg K. H. Madsen

**Affiliations:** † Institute of Materials Chemistry, Technical University of Vienna, Getreidemarkt 9/165-TC, A-1060 Vienna, Austria; ‡ Institute for Information Systems Engineering, Technical University of Vienna, Favoritenstrasse 9-11/194, A-1040 Vienna, Austria

## Abstract

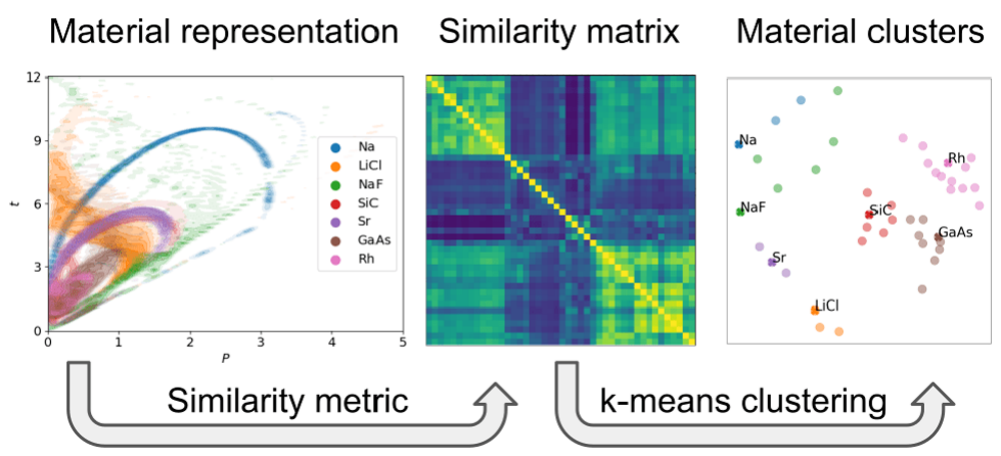

Benchmarking DFT functionals is complicated since the results highly depend on which properties and materials were used in the process. Unwanted biases can be introduced if a data set contains too many examples of very similar materials. We show that a clustering based on the distribution of density gradient and kinetic energy density is able to identify groups of chemically distinct solids. We then propose a method to create smaller data sets or rebalance existing data sets in a way that no region of the meta-GGA descriptor space is overrepresented, yet the new data set reproduces average errors of the original set as closely as possible. We apply the method to an existing set of 44 inorganic solids and suggest a representative set of seven solids. The representative sets generated with this method can be used to make more general benchmarks or to train new functionals.

## 1 Introduction

Currently, the most widely used theoretical method to predict the different properties of materials is Kohn–Sham-density functional theory (KS-DFT).^1^ The accuracy of this approach mainly depends on the underlying functional for the exchange-correlation energy, *E*_xc_. To compare and rank these functionals, various benchmarks were done on different data sets and properties. Notable data sets for molecules are the G2/97^2^ and G3/99^3^ containing 302 and 376 energies (atomization- and ionization energies, proton- and electron affinities, and reaction barrier heights), respectively. Similar databases are used to benchmark functionals for solids as well, like a set^4^ of 18 solids of different types (main group metals, ionic solids, semiconductors, and transition metals), an extension of this set containing 44 strongly bound solids^5^ or a set of more than 300 materials used to benchmark the SCAN functional.^6^ Yet these benchmark data sets are often based on “what is available”. This can potentially introduce biases for types of materials which are either over- or underrepresented. Unbalanced data sets are problematic and test results can depend on the chosen set in a way that is not transparent. Furthermore, compounds which are very similar and provide little new information lead to unnecessary computational effort.

To avoid or make bias more transparent and for computational efficiency, it would be appealing to create smaller representative benchmark data sets. Still, the literature on this is surprisingly scarce. One approach created two data sets for molecules containing six representative atomization energies and barrier heights, respectively.^7^ The results are quite appealing. Obviously from the point of view of computational effort but also because the representative molecules are both diverse and make sense as representatives of the much larger original data sets. As such, finding representative molecules is also interesting as a data-driven approach to developing a chemical intuition. On the other hand, the representative molecules were chosen to best possibly reproduce the average errors obtained for the complete data sets.^7^ Thereby bias in the original data set will tend to be reflected in the representative set. A group of compounds that are strongly represented in the original data set will also tend to be in the representative set. Furthermore, small, but unique, groups of compounds could be left out, thereby potentially covering up problems of a given functional. In this respect, we recently analyzed how the SCAN functional, which generally performs well for lattice parameter calculations, fails for alkali metals.^8^ As there are only a limited number of alkali metals, large errors for this small group are not punished in the benchmarks.

In the present study, we aim to find materials which are both representative in terms of the electron density distributions sampled and in terms of the errors. Our approach is based on clustering materials according to their density distribution. The idea being that the materials are clustered according to what part of parameter space, in this case density gradients and kinetic energy densities, they occupy. Then representative materials are chosen according to their errors.

## 2 Methodology

### 2.1 Density Representation and Metric

To achieve the clustering, we need a descriptor for the materials on which we can define a similarity function. Since the differences between the functionals arise from the different functional forms for *E*_xc_ energy, it seems natural to base our descriptors on the quantities which enter these. The most common functionals for solids are semilocal, where *E*_xc_ is given as a functional of the density, *n*, the magnitude of the density gradient, |∇*n*|, and sometimes the Laplacian of the density and the kinetic energy density (KED) τ defined as
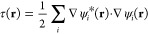
1The different levels of approximations use different arguments. The local density approximation (LDA) uses only the density, the generalized gradient approximations (GGAs) use the gradients as well, and meta-GGAs (mGGAs) can use all four parameters. In the present study, we focus on functionals and descriptors based on *n*, |∇*n*|, and τ. We also tested including the Laplacian in our descriptors, but in agreement with our earlier findings,^9^ we did not find important differences in the results and it is left out in the following discussion.

Semilocal functions are typically written in terms of the LDA and an enhancement factor which depends on normalized dimensionless, or reduced, values of the mentioned quantities. It is in this enhancement factor that the functionals typically differ. We use the reduced quantities
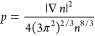
2
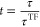
3as the descriptors. Here τ^TF^ = (3/10)(3π^2^)^2/3^*n*^5/3^ is the Thomas–Fermi KED.

We consider the 44 solids in a previously published data set.^5^ We use the all-electron KS-DFT code WIEN2k^10,11^ and the PBE functional to calculate the density values. Based on these data, *p*–*t* maps for each material are created by binning the densities in a mesh of *p*–*t* combinations with a bin width of 0.02 in both directions. The core regions of the atoms contain a large number of points with large values of electron density and low values of the reduced quantities, eqs 2 and 3. To avoid that these chemically inactive regions dominate the descriptors, the mesh was subsequently turned into an indicator function being 1 if there was at least one point at the given *p*,*t* value and 0 otherwise. After this, a Gaussian smearing was applied to the map with a standard deviation of 0.06.

The choice of the similarity/distance metric is essential to achieve a good clustering. Since our goal is to find materials which cover the same region of the *p*–*t* space, if two materials cover overlapping regions their distance should be close to zero. The more specific requirement when defining the distance is that it should have a maximum of one, when the materials have no overlap and should not diverge based on the exact shapes of the occupied regions. Therefore, simple Euclidean distances between the matrices are not usable in this case. A choice for similarity which obeys the mentioned requirements is the normalized dot product of the maps, defined the following way:
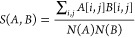
4where *A* and *B* represent the *p*–*t* maps of two given materials and *i*,*j* are the index bins of *p* and *t*. The *N* normalization function is
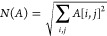
5The values of *S* are always between 0 and 1, being 0 when there is no overlap in the density maps, and 1 when the maps match exactly. Using this, we can define a distance function simply as 1 – *S*(*A*,*B*).

### 2.2 Clustering Method

The clustering is done using *k*-means clustering, more specifically Lloyd’s algorithm.^12^ Given *N* samples, every sample being a *d* dimensional vector, and the desired number of clusters, *C*, the algorithm chooses *C* samples randomly as cluster centers. Then two steps are iterated until convergence. First, every sample is assigned to the cluster which has the closest centroid. Second, the positions of the centroids are updated to the mean of the samples of the given cluster. With this setup, the algorithm is guaranteed to converge to a minimum sum of squared distances between the samples and their cluster centers.

Since the basic *k*-means algorithm works in Euclidean spaces, our distance matrix has to be embedded in a *d*-dimensional Euclidean space. For this, the multidimensional scaling (MDS)^13^ technique is used, which places the materials in a *d*-dimensional space based on the distance matrix in a way that the Euclidean distances between their locations fit the distance matrix as well as possible. The dimensionality of the embedding space limits the achievable accuracy of the MDS, so we opted to use the maximum number of dimensions to represent our data. For our 44 data points, there are 43 dimensions, since any higher dimensional embedding can be reduced to 43 dimensions. This embedding method resulted in a 0.02 average absolute error between the distance matrix based on the similarity defined in eq 4 and the Euclidean distance matrix of the embedded materials.

Because both the MDS and the *k*-means algorithm involves some randomness, we evaluated multiple different embedding and ran the *k*-means algorithm 10 000 times with random starting centroids for every embedding. We will later focus on seven clusters (*C* = 7). These clusters and especially the representative sets based on these were very stable across multiple runs. The small differences in the loosely connected clusters are discussed later. These clusters were also compared to results from affinity propagation or *k*-means clusterings on the L1 distances of normalized density maps and the resulting clusters are not only consistent with respect to the random seeds but also across different clustering methods.

### 2.3 Error Based Representative Sets

We also apply the method that was used to generate the AE6 and BH6 sets.^7^ The method aims to choose a smaller subset of the original data, which reproduces the mean signed error (MSE), mean unsigned error (MUE), and root mean squared error (RMSE) as well as possible. If we denote the difference between, e.g., the MSE of the entire database and the representative set when using functional *i* as Δ_MSE_(*i*), then the aim is the minimization of the root mean squared deviation (RMSD), defined as
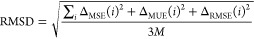
6where *M* represents the number of different functionals. To evaluate how good a representative set is, the percent error in representation (PEIR) was used:
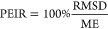
7where ME is the mean error:
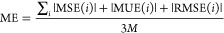
8with the errors calculated on the whole data set. When the whole database is used as representative set, then the PEIR value is zero.

In our case, the database consists of 44 materials and we have 24 different GGA and mGGA functionals for three different properties (lattice parameter, bulk modulus, and cohesive energy). To find a representative set with *N* materials, the PEIRs for the three properties are calculated for all  combinations, and the one with the lowest average PEIR is chosen. A direct minimization of the PEIR by choosing 7 compounds from the entire 44 compounds results in the group of
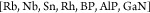
setPEIRwith a PEIR of 15%. This set inherently carries the imbalances of the full set. Six of the seven compounds belong to the transition metals and diamond-lattice semiconductors. It only contains one representative of the alkali metals and none of the ionic materials nor the alkaline earth metals, which are chemically distinct groups and should be present in a small set. As will be discussed later, setPEIR fails to sample a variety of densities and can, even if it reproduces average errors well, somewhat misrepresent the error for a specific functional.

## 3 Results and Discussion

**Figure 1:**
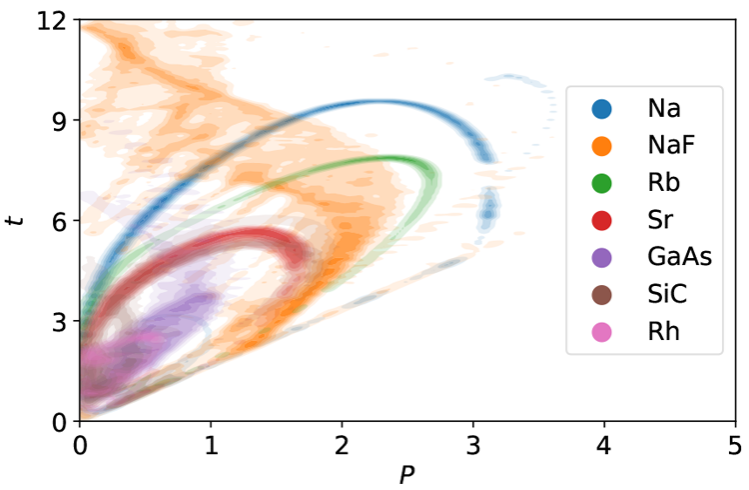
*p*–*t* maps of seven representative solids. The clear difference between the different colored regions show that chemically different materials sample distinct regions of the *p*–*t* space.

Considering first the *p*–*t* maps as descriptors on which the clustering should be based, they are shown for seven different compounds in Figure 1. It can be seen that these chemically distinct compounds also sample different regions of the *p*–*t* maps. Changing, e.g., the dependence of the *E*_xc_ functional on the high *p*–high *t* region would mainly influence the results obtained for Na, NaF, and similar materials, whereas it would hardly influence the results obtained for the close-packed metal Rh or the semiconductor GaAs. This difference between alkali metals and d-metals or semiconductors falls in line with earlier studies. It has previously been noticed for the atomic electron densities where the maximum value of *p* (not counting the diverging tail far from the nuclei) decreases along the rows and also along the columns of the periodic table.^14^ Furthermore, in the case of solid Si and LiF, regions around the outer shell of Li were found to have twice as large *p* values as in Si.^15^ The empty space of the bottom right part of Figure 1 illustrates the von Weizsäcker limit (*t* > 5*p*/3). The distance on the *y* axis from this limit is called α = *t* – 5*p*/3 and has been shown to carry important information about the bonding properties. In regions occupied by a single orbital α = 0,^16^ while in regions with slowly varying density α ≈ 1.^17^ α has been also shown to take low values in the covalent bonds of graphite, while being much larger in the interlayer region.^18^

**Figure 2:**
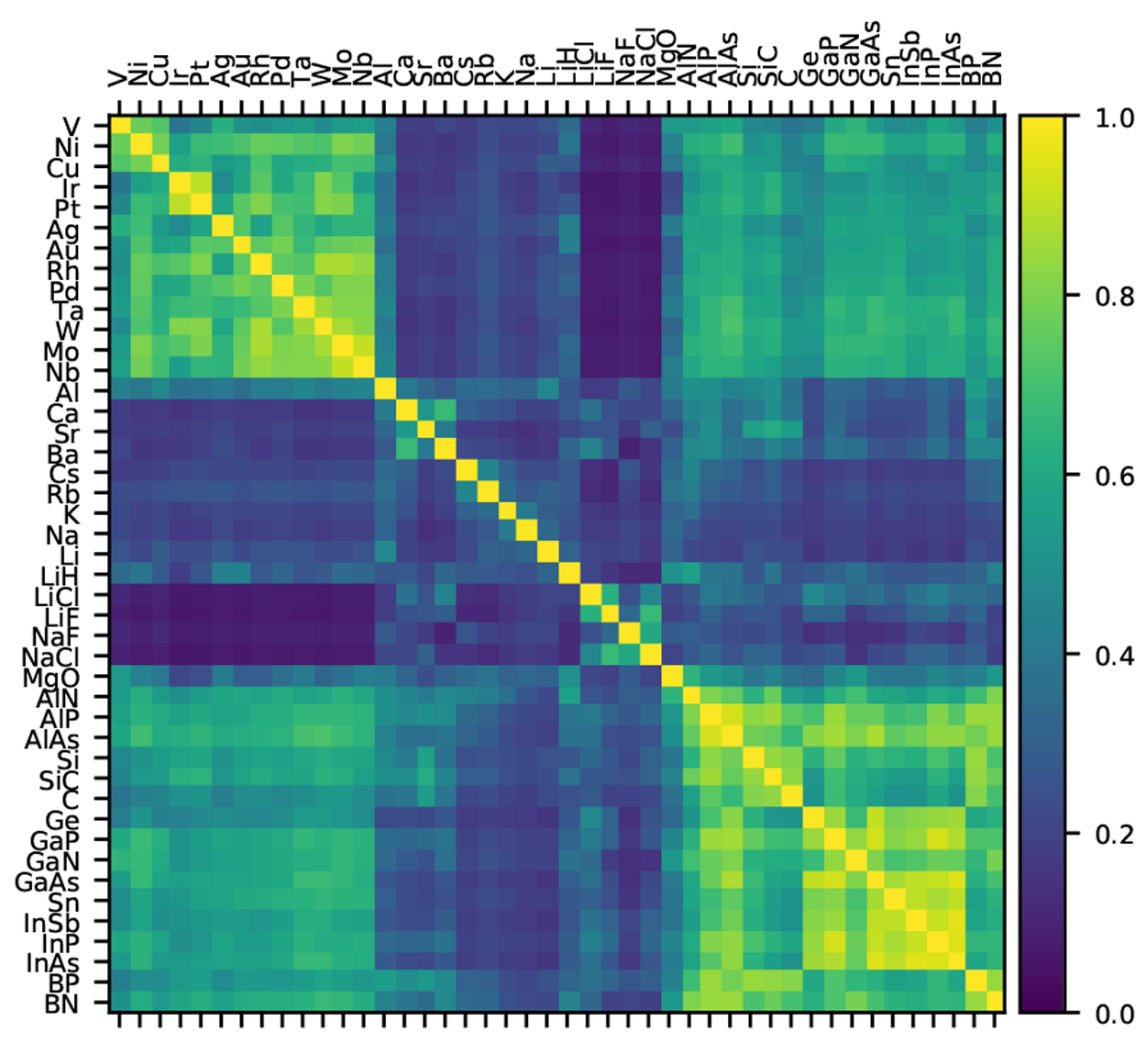
Similarity matrix between materials, with the metal cluster on the top left, the semiconductor cluster on the bottom right, and the less similar groups of ionic materials and alkali- and alkaline earth metals in the middle.

The similarity matrix, eq 4, of the 44 materials considered here is shown in Figure 2. The materials are in an ad-hoc order based on intuition. However, we can still identify multiple groups of similar compounds. These are the close-packed metals, top left, and the semiconductors, bottom right. Some similarity can also be seen between some of the ionic bound materials.

**Figure 3:**
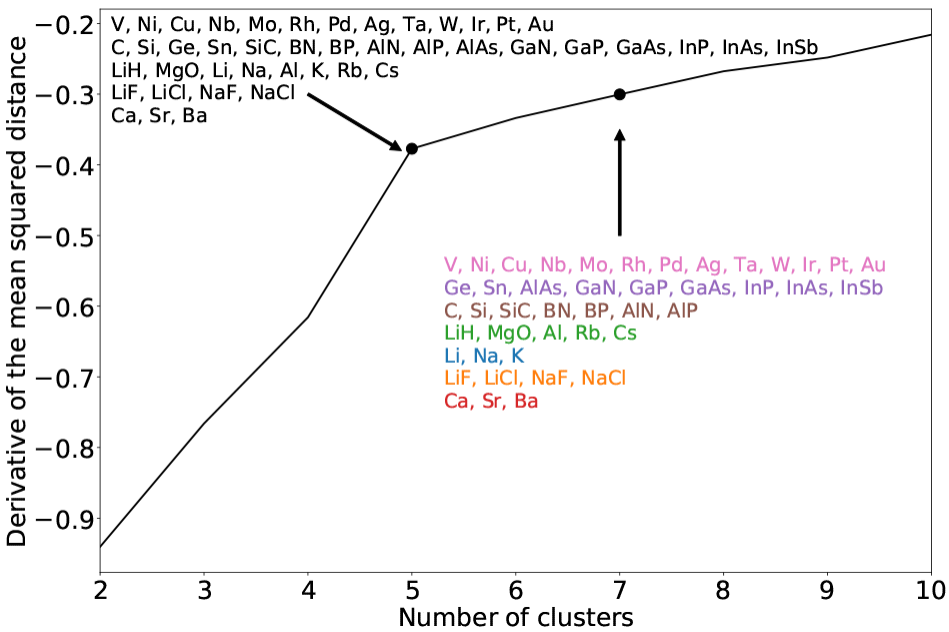
Derivative of the average squared intracluster distances with respect to number of clusters. The colors correspond to the cluster colors of Figures 1 and 4.

To find the optimal number of clusters, we ran the *k*-means clustering for up to 10 clusters. The derivative of the average squared intracluster distances are shown in Figure 3. It can be seen that making more than seven clusters does not improve the grouping significantly. The seven clusters formed this way are [V, Ni, Cu, Nb, Mo, Rh, Pd, Ag, Ta, W, Ir, Pt, Au], [C, Si, SiC, BN, BP, AlN, AlP], [Ge, Sn, AlAs, GaN, GaP, GaAs, InP, InAs, InSb], [LiH, MgO, Al, Rb, Cs], [LiF, LiCl, NaF, NaCl], [Ca, Sr, Ba], and [Li, Na, K]. The intuitive groups that could be recognized by visual inspection of Figure 2 can be found in this clustering as well. It is pleasing that the transition metals form one large cluster. The diamond-lattice semiconductors are split into two relatively large clusters. Figure 3 shows how the semiconductors would be grouped into one cluster if only five clusters should be made. The improvement in mean-squared distance between five and seven clusters is however substantial, and the splitting is also systematic in the sense that one diamond-lattice cluster tends to contain the atoms from the early periods of the periodic table and the other cluster the atoms from the later periods. There are further smaller clusters of ionic, alkali-, and alkaline earth metals. One cluster contains a mixture of ionic compounds and metals, which is also the most unstable cluster, splitting in [LiH, MgO, Al] and [Rb, Cs] groups when eight instead of seven clusters are formed.

**Figure 4:**
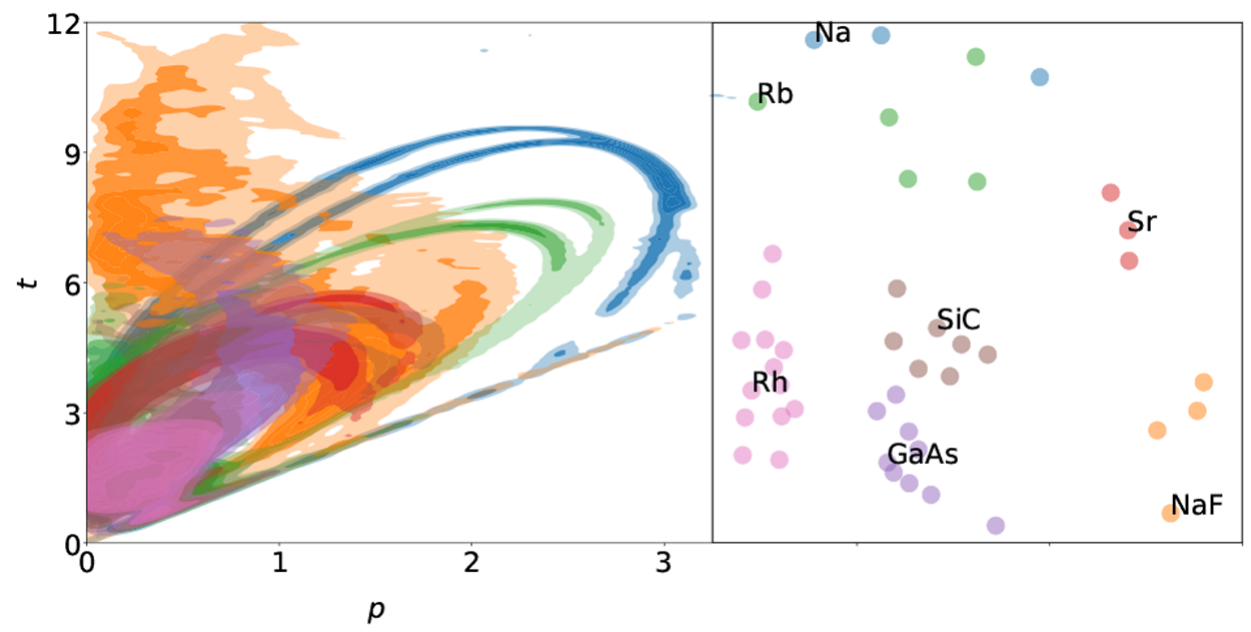
(left) *p*–*t* maps of the “average materials” of the seven clusters. The colors are chosen such that they agree with the clusters in Figure 3 and their representative materials in Figure 1. (right) The 2D representation generated by placing the materials in such a way that the Euclidean distances between their locations fit the distance matrix as well as possible according to the multidimensional scaling algorithm.^13^ The points are colored according to the clusters formed by the *k*-means clustering in the 43D space and marked by the representative material.

The right panel of Figure 4 shows the 2D representation of the distance matrix generated by the MDS algorithm. The materials are colored according to the clustering in the 43D space. Each cluster is labeled by one solid, which will later be identified as its first representative. The illustration highlights the strong similarity inside the metal (pink) and semiconductor (purple, brown) clusters and the lower similarity of the clusters containing ionic compounds and alkali and alkaline earth metals. Using only the 2D representation introduces some artifacts mostly around the Na and Rb clusters which results in some of their elements to be seemingly assigned to the wrong cluster. This is only caused by the mismatch of the 2D and 43D representations. The left part of Figure 4 shows the *p*–*t* map obtained by averaging over the solids in each of the seven groups thereby illustrating the most significant regions of *p*–*t* values for every cluster. These “average materials” highlight different regions of mGGA functionals sampled by the materials. If one would use the representative set predicted by the naive PEIR minimization method, setPEIR, the blue, orange, and red regions would be unsampled, and six of the seven materials would come from the pink, brown, and purple areas. These three areas include only the semiconductor and metal clusters and are constrained to the relatively low *p*–*t* regions.

Having the seven clusters, we tested two approaches to find representative sets. The first approach was to calculate the PEIR for every possible combination of seven materials where each material must be from a separate cluster and choose the set with lowest PEIR. This constrained optimization results in the set
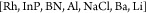
setAwith a PEIR of 21%. While this PEIR value is obviously higher than the value of 15% obtained for setPEIR, setA seems more representative. Not only in terms of the *p*–*t* maps but also intuitively, in that it is much more diverse in terms of chemistry.

The second approach avoids optimizing the PEIR with respect to the entire data set and instead chooses from each cluster the material which represents its own cluster best, i.e., the material from each cluster which gives the smallest PEIR with respect to its own cluster. The set formed this way is
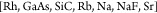
set1Again this set is representative in terms of *p*–*t* and chemical intuition. This set has not been chosen to minimize the PEIR, and the resulting PEIR of 38% is substantially higher than for the sets setPEIR and setA formed by minimizing the total PEIR. However, our goal is not to reproduce the average errors of the full set exactly but to sample as vast regions of the phase space as possible without unreasonably deviating from the average errors. In the end, set1 is preferred since the optimization minimizes the impact of the inbalances of the original data set. These seven materials are the ones used to label the clusters in Figure 4, and they were used to exemplify *p*–*t* maps in Figure 1. The strong similarity between Figure 1 and Figure 4 shows that the representatives indeed sample the same region as the “average materials” of the given clusters.

**Figure 5:**
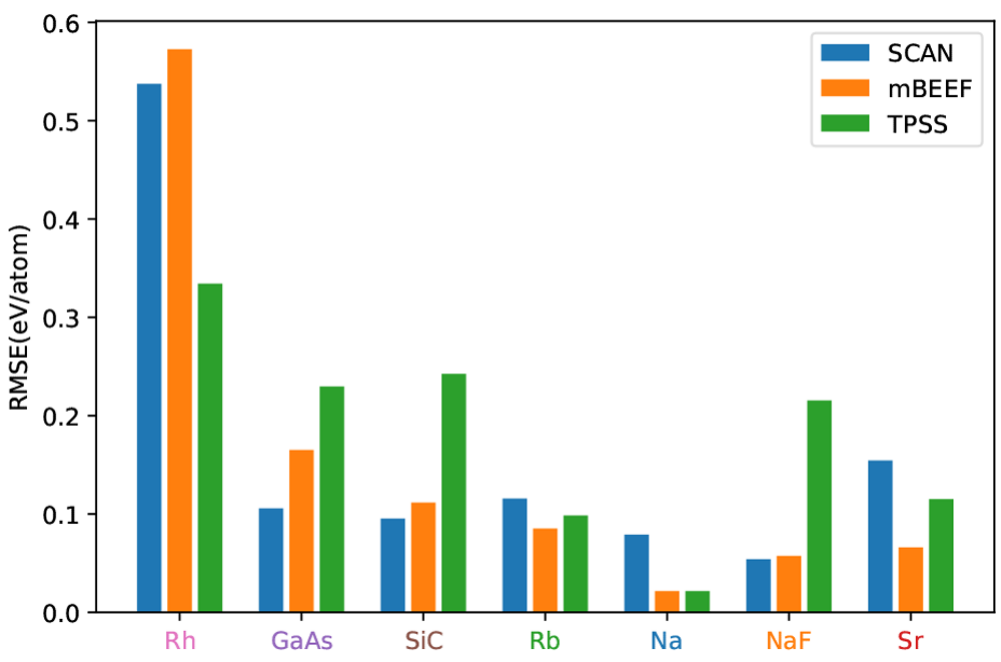
Cohesive energy RMSE of the seven clusters using the three analyzed functionals. The largest errors for the SCAN and mBEEF functionals can always be found in the transition metal cluster, while for TPSS the semiconductor clusters also show large errors.

**Figure 6:**
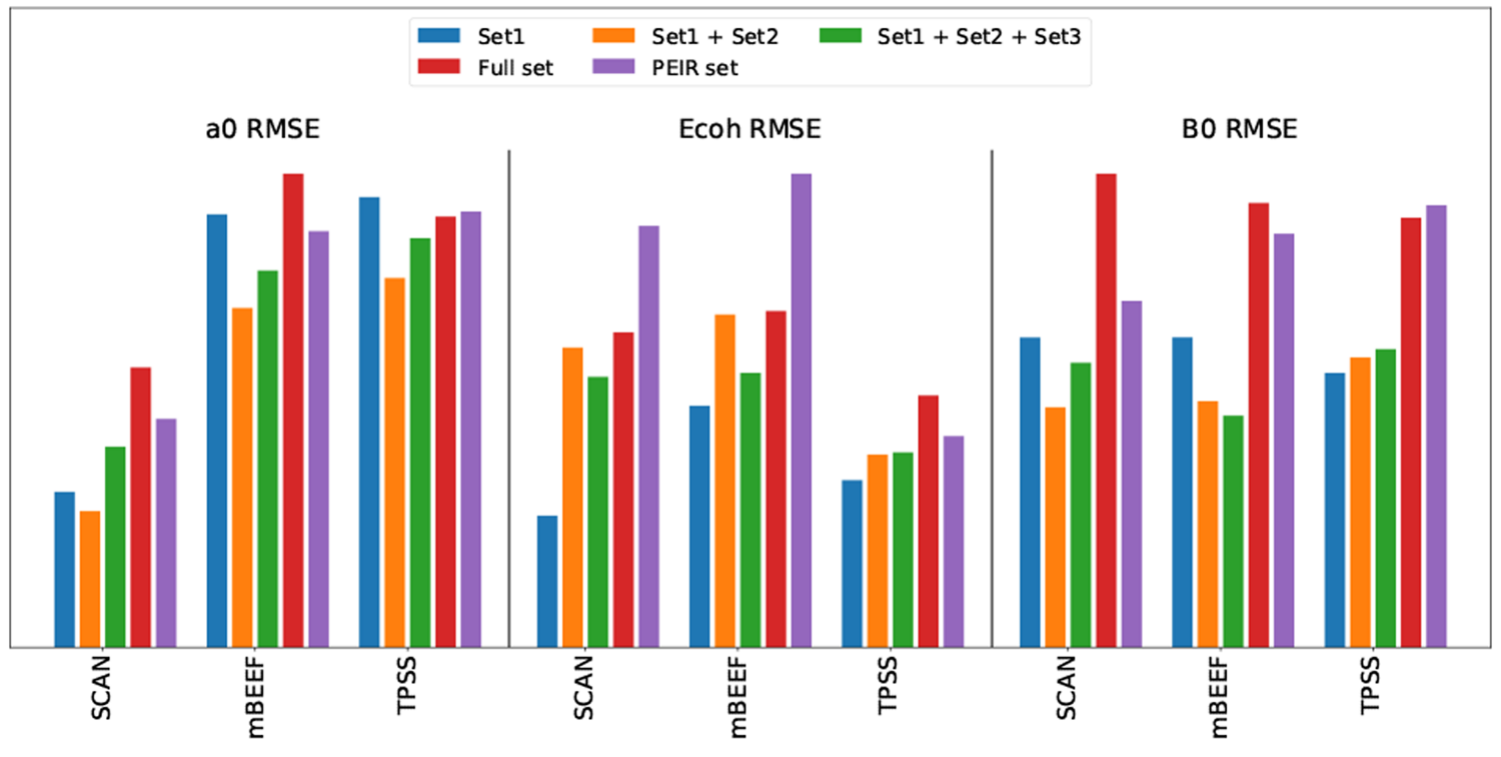
RMSE of three mGGAs for the lattice parameter (left), cohesive energy (middle), and bulk modulus (right) calculated on the original full database, the seven materials set minimizing the PEIR, and three different size representative sets. The larger representative sets are extended versions of the smaller ones, including two and three materials from every cluster. The errors are scaled, so for every property, maximum errors have the same height.

Even with representative sets optimized to with the best possibility to reproduce an error averaged over functionals and properties according to eq 6, it is an open question how well the error for a given property and for a given functional is represented. In Figure 6, we have chosen the three functionals SCAN,^19^ TPSS,^20^ and mBEEF^21^ and show the specific RMSE of setPEIR, setA, and set1 for the three properties. As expected, none of the representative sets exactly reproduces the average errors of the entire set. It is worth noting that setPEIR, which was optimized to minimize PEIR without constraints, can result in errors which differ substantially from the full set, e.g., for the cohesive energies obtained with mBEEF or SCAN. It is also noticeable that both sets based on *p*–*t* clustering almost always give a lower RMSE than the full set. This is partially caused by the balancing of the data set. The cohesive energy errors of the seven clusters with the three functionals are shown in Figure 5. The cluster of close packed metals has the highest RMSE for the SCAN and mBEEF functionals, while for TPSS the two semiconductor clusters also show comparable errors. In the representative sets, these three clusters are down-weighted, since they contain many compounds sampling the same regions of the *p*–*t* space, and therefore the overall errors in the representative sets are reduced.

These results also illustrate that picking one representative material for each cluster may not always be adequate. For both setA and set1, Rh was picked to represent the metal cluster. However, Rh has an error in *E*_coh_ of 0.3 eV/atom when using the SCAN functional, whereas the RMSE for cohesive energies of the transition metal cluster is 0.54 eV/atom for SCAN. So while Rh is the best material to represent the average error of multiple different functionals, in the sense of eq 6, it is somewhat misleading for the *E*_coh_ error of SCAN. Consequently both set1 and setA give to some degree artificially low error for the SCAN cohesive energy, see Figure 6. The sets formed by choosing from the representative clustering can, however, be systematically improved by extending the groups of representative materials with additional elements of the clusters. If we choose one additional solid from each cluster by minimizing the RMSD with respect to that cluster, we obtain
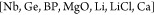
set2Using set1 and set2 as representative materials, a systematic improvement can be observed, see Figure 6. This can be continued by extending with a third set
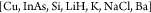
set3The three clusters containing just three compounds, Figure 3, are then fully present. If the computational cost of the functional evaluation is not a concern, our approach can be still useful to balance the data set, simply by weighting the different materials based on their cluster size. As an example, the error bar on *E*_coh_ using TPSS seems to be overestimated due to the strong weight of the transition metal cluster which only samples a rather small part of the *p*–*t* space.

**Figure 7:**
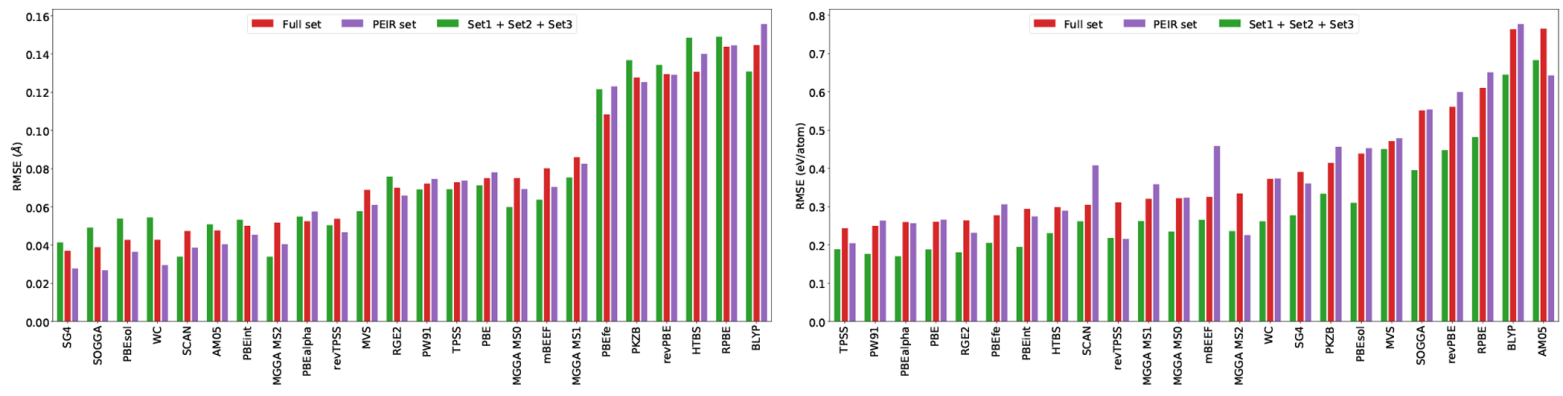
RMSE of 24 GGAs and mGGAs for the lattice parameter (upper panel) and cohesive energy (lower panel) calculated on the original full database, the seven materials set minimizing the PEIR, and the larger representative set that includes three materials from every cluster. The functionals are ordered according to the RMSE obtained with the original full database. The references for all of the functionals can be found in ref (5).

Irrespective of the average errors of the original set and a representative set, the ranking of the functionals in terms of accuracy is also important. Figure 7 shows the RMSE of 24 GGA and mGGA functionals for the lattice parameter and cohesive energy. The functionals are ordered according to the RMSE of the original full set. The ranking of the functionals with two other sets (setPEIR and the set including three materials from every cluster) shows similarities with the original set. By splitting the functionals into three groups, the most accurate, the least accurate, and the middle ones, the groups remain more or less the same independent of the set. There can be inversions within a group of functionals compared to the original database. As discussed above, the error on the cohesive energy seems overestimated for mBEEF and SCAN when using the setPEIR. The representative sets on the other hand give a lower average error for the lattice constants with SCAN and MS2 functionals, mainly due to the down weighting of the transition metals.

## 4 Summary and Conclusions

In the current study, we presented a way to group different inorganic solids based on their electron density, allowing us to identify solids which are sampling the same regions of *p*–*t* descriptor space. To achieve the grouping, we defined a distance metric, which is bound between 0 and 1, and represents the dissimilarities of the previously mentioned descriptors of different materials. Using multidimensional scaling and *k*-means clustering, we formed clusters of similar materials. These are not a pure mathematical construction but also reflect basic chemical properties. Based on the clustering, a small representative set of bulk solid materials is constructed, which not only samples as big regions of the *p*–*t* space as possible but also aims to reproduce the average errors of the original data set for multiple GGA and mGGA functionals.

The smaller representative sets of the original database allow for faster evaluation of GGA and mGGA functionals. As the method is able to identify materials which occupy similar regions of the *p*–*t* space, thus down weighting highly populated areas can lead to a more general evaluation or functional training.

More recently it has become possible to create test databases based on higher level ab initio methods.^22^ An important advantage of the clustering is that it allows for a screening of compounds based just on the DFT descriptors before computationally heavy calculations are performed.

## References

[ref1] Kohn W.; Sham L. J. Self-Consistent Equations Including Exchange and Correlation Effects. Phys. Rev. 1965, 140, A1133–A1138. 10.1103/PhysRev.140.A1133.

[ref2] Curtiss L. A.; Raghavachari K.; Redfern P. C.; Pople J. A. Assessment of Gaussian-2 and density functional theories for the computation of enthalpies of formation. J. Chem. Phys. 1997, 106, 1063–1079. 10.1063/1.473182.

[ref3] Curtiss L. A.; Raghavachari K.; Redfern P. C.; Pople J. A. Assessment of Gaussian-3 and density functional theories for a larger experimental test set. J. Chem. Phys. 2000, 112, 7374–7383. 10.1063/1.481336. 16392475

[ref4] Staroverov V. N.; Scuseria G. E.; Tao J.; Perdew J. P. Tests of a ladder of density functionals for bulk solids and surfaces. Phys. Rev. B: Condens. Matter Mater. Phys. 2004, 69, 075102 10.1103/PhysRevB.69.075102.

[ref5] Tran F.; Stelzl J.; Blaha P. Rungs 1 to 4 of DFT Jacob’s ladder: Extensive test on the lattice constant, bulk modulus, and cohesive energy of solids. J. Chem. Phys. 2016, 144, 204120 10.1063/1.4948636. 27250292

[ref6] Zhang Y.; Kitchaev D. A.; Yang J.; Chen T.; Dacek S. T.; Sarmiento-Pérez R. A.; Marques M. A. L.; Peng H.; Ceder G.; Perdew J. P.; Sun J. Efficient first-principles prediction of solid stability: Towards chemical accuracy. npj Comput. Mater. 2018, 4, 9 10.1038/s41524-018-0065-z.

[ref7] Lynch B. J.; Truhlar D. G. Small Representative Benchmarks for Thermochemical Calculations. J. Phys. Chem. A 2003, 107, 8996–8999. 10.1021/jp035287b.

[ref8] Kovács P.; Tran F.; Blaha P.; Madsen G. K. H. Comparative study of the PBE and SCAN functionals: The particular case of alkali metals. J. Chem. Phys. 2019, 150, 164119 10.1063/1.5092748. 31042906

[ref9] Tran F.; Kovács P.; Kalantari L.; Madsen G. K. H.; Blaha P. Orbital-free approximations to the kinetic-energy density in exchange-correlation MGGA functionals: Tests on solids. J. Chem. Phys. 2018, 149, 144105 10.1063/1.5048907. 30316291

[ref10] Blaha P.; Schwarz K.; Madsen G. K. H.; Kvasnicka D.; Luitz J.; Laskowski R.; Tran F.; Marks L. D. WIEN2k: An Augmented Plane Wave plus Local Orbitals Program for Calculating Crystal Properties; Vienna University of Technology: Vienna, Austria, 2018.

[ref11] Blaha P.; Schwarz K.; Tran F.; Laskowski R.; Madsen G. K. H.; Marks L. D. WIEN2k: An APW+lo program for calculating the properties of solids. J. Chem. Phys. 2020, 152, 074101 10.1063/1.5143061. 32087668

[ref12] Lloyd S. Least squares quantization in PCM. IEEE Trans. Inf. Theory 1982, 28, 129–137. 10.1109/TIT.1982.1056489.

[ref13] Kruskal J. B. Multidimensional scaling by optimizing goodness of fit to a nonmetric hypothesis. Psychometrika 1964, 29, 1–27. 10.1007/BF02289565.

[ref14] del Campo J. M.; Gázquez J. L.; Alvarez-Mendez R. J.; Vela A. The reduced density gradient in atoms. Int. J. Quantum Chem. 2012, 112, 3594–3598. 10.1002/qua.24241.

[ref15] Haas P.; Tran F.; Blaha P.; Schwarz K.; Laskowski R. Insight into the performance of GGA functionals for solid-state calculations. Phys. Rev. B: Condens. Matter Mater. Phys. 2009, 80, 195109 10.1103/PhysRevB.80.195109.

[ref16] Becke A. D.; Edgecombe K. E. J. Chem. Phys. 1990, 92, 5397–5403. 10.1063/1.458517.

[ref17] Sun J.; Xiao B.; Fang Y.; Haunschild R.; Hao P.; Ruzsinszky A.; Csonka G. I.; Scuseria G. E.; Perdew J. P. Density Functionals that Recognize Covalent, Metallic, and Weak Bonds. Phys. Rev. Lett. 2013, 111, 106401 10.1103/PhysRevLett.111.106401. 25166685

[ref18] Madsen G. K. H.; Ferrighi L.; Hammer B. Treatment of Layered Structures Using a Semilocal meta-GGA Density Functional. J. Phys. Chem. Lett. 2010, 1, 515–519. 10.1021/jz9002422.

[ref19] Sun J.; Ruzsinszky A.; Perdew J. P. Strongly Constrained and Appropriately Normed Semilocal Density Functional. Phys. Rev. Lett. 2015, 115, 036402 10.1103/PhysRevLett.115.036402. 26230809

[ref20] Tao J.; Perdew J. P.; Staroverov V. N.; Scuseria G. E. Climbing the Density Functional Ladder: Nonempirical Meta-Generalized Gradient Approximation Designed for Molecules and Solids. Phys. Rev. Lett. 2003, 91, 146401 10.1103/PhysRevLett.91.146401. 14611541

[ref21] Wellendorff J.; Lundgaard K. T.; Jacobsen K. W.; Bligaard T. mBEEF: An accurate semi-local Bayesian error estimation density functional. J. Chem. Phys. 2014, 140, 144107 10.1063/1.4870397. 24735288

[ref22] Schmidt P. S.; Thygesen K. S. Benchmark Database of Transition Metal Surface and Adsorption Energies from Many-Body Perturbation Theory. J. Phys. Chem. C 2018, 122, 4381–4390. 10.1021/acs.jpcc.7b12258.

